# Real-world survival outcomes of wedge resection versus lobectomy for cT1a/b cN0 cM0 non-small cell lung cancer: a single center retrospective analysis

**DOI:** 10.3389/fonc.2023.1226429

**Published:** 2023-08-17

**Authors:** Luca Bertolaccini, Andrea Cara, Matteo Chiari, Cristina Diotti, Nimrod Glick, Shehab Mohamed, Clarissa Uslenghi, Antonio Mazzella, Daniela Brambilla, Raffaella Bertolotti, Giulia Sedda, Lorenzo Spaggiari

**Affiliations:** ^1^ Department of Thoracic Surgery, IEO, European Institute of Oncology IRCCS, Milan, Italy; ^2^ Department of Oncology and Hemato-Oncology, University of Milan, Milan, Italy

**Keywords:** wedge resections, lobectomies, lung cancer, outcomes, survival analysis, multivariable analysis

## Abstract

**Background:**

JCOG0802/WJOG4607L showed benefits in overall survival (OS) of segmentectomy. CALGB 140503 confirmed that sublobar resection was not inferior to lobectomy concerning recurrence-free survival (RFS) but did not provide specific OS and RFS according to the techniques of sublobar resections. Hence, we retrospectively analyze the survival differences between wedge resection and lobectomies for stage IA lung cancer.

**Methods:**

We reviewed the clinical records of patients with clinical stage IA NSCLC over 20 years. The inclusion criteria were: preoperative staging with CT scan and whole body CT/PET; tumor size <20 mm; wedge resections or lobectomies with or without lymph node dissection; NSCLC as the only primary tumor during the follow-up period. We excluded: multiple invasive lung cancer; positive resection margin; preoperative evidence of nodal disease; distant metastasis at presentation; follow-up time <5 years. The reverse Kaplan – Meier method estimated the median OS and PFS and compared them by the log-rank test. The stratified backward stepwise Cox regression model was employed for multivariable survival analyses.

**Results:**

539 patients were identified: 476 (88.3%) lobectomies and 63 (11.7%) wedge resections. The median OS time for the whole cohort was 189.7 months (range: 173.7 – 213.9 months). The 5-year wedge resection and lobectomy OS were 82.2% and 87.0%. The 5-year RFS of wedge resection and lobectomy were 17.8% and 28.9%. The log-rank test showed no significant differences (p = 0.39) between wedge resections and lobectomies regarding OS and RFS (p = 0.23).

**Conclusions:**

Lobectomy and wedge resection are equivalent oncologic treatments for individuals with cN0/cM0 stage IA NSCLC <20 mm. Validating the current findings requires a prospective, randomized comparison between wedge resection and standard lobectomy to establish the prognostic significance of wedge resection.

## Introduction

Surgery is the cornerstone approach for resectable, early-stage non-small cell lung cancer (NSCLC) (1). Twenty-six years ago, the North American Lung Cancer Study Group revealed superior overall survival (OS) after lobectomy than the wedge or segmental resection for early-stage NSCLC (2). Last year, the Japanese randomized control trial JCOG0802/WJOG4607L was the first phase 3 trial published that showed the benefits in OS of segmentectomy versus lobectomy (3). This year the CALGB 140503, a multicenter, international, randomized, non-inferiority, phase 3 trial involving patients with peripheral NSCLC <2 cm, confirmed that sublobar resection was not inferior to lobectomy concerning recurrence-free survival (RFS). OS was similar to the two procedures (4). Nevertheless, CALGB 140503 was based on 357 lobectomies and 340 sublobar resections (59.1% wedge resection versus 37.9% anatomical segmentectomy). CALGB 140503 does not provide specific OS and RFS according to the different sublobar resections techniques (5).

Given the urgency of this health policy question and the results of the two contemporary prospective trials (3, 4) currently available, we examined in a retrospective analysis of a highly selected population the differences in survival between wedge resection and lobectomies for stage IA lung cancer.

## Material and methods

The Ethics Committee and the Internal Review Board, informed of the database extraction, did not require approval because of the study’s retrospective nature. Before surgery or medical treatment, written authorization was obtained by patients at the time of hospital admission to use their personal information for therapeutic purposes and separately for epidemiologic research investigations. This manuscript was written according to the Strengthening the Reporting of Cohort Studies in Surgery (STROCSS) Statement (6). The STROCSS checklist is available as **Supplemental File 1**.

We reviewed clinical records of patients who received curative surgeries for clinical stage IA NSCLC at our institution over 20 years (1998 – 2017).

We identified the cohort of patients using the following inclusion criteria:

- Preoperative staging with chest and abdomen CT scan and whole body 18-fluorine-fluorodeoxyglucose CT/positron-emission-tomography (18F-FDG CT/PET).
- Tumor size <20 mm.
- Wedge resections or lobectomies with or without lymph node dissection.
- NSCLC was the only primary tumor during the follow-up period. We excluded patients using the following criteria:
- Multiple invasive lung cancer.
- Positive resection margin.
- Preoperative evidence of nodal disease.
- Distant metastasis at presentation.
- Follow-up time <5 years.

Lobectomy with systemic lymph node dissection was our primary procedure, and all patients were clinical N0 before the operation (minor axis of lymph node ≤1 cm on thin-section CT image). The surgical operations were done with a minimally invasive (Robotic or Video-Assisted) or thoracotomic approach. Wedge resection was performed in elderly patients or compromised cardiopulmonary function or for the patients’ willingness. A resection margin of at least 1 cm was secured in wedge resections and recorded in the pathological reports. Margin recurrence was defined as tumor recurrence at the original surgical margin. The surgical margin is identified mainly by the metal shadow of the stapled line on the CT scan image, and the identification of recurrence was confirmed by 18F-FDG CT/PET or pathological biopsy. All cases were discussed at multidisciplinary tumor boards for surgical indication as well as for the treatment strategy.

All the patients received regular postoperative follow-up examinations. Further evaluation was performed when recurrence or metastasis was suspected, including a CT scan or 18F-FDG CT/PET scan and brain magnetic resonance. Local recurrence was defined as an occurrence within the ipsilateral hemithorax, including the lung, lymph node, and pleura, and distant recurrence was defined as distant organ metastases. In this study, the outcomes of interest included RFS and OS.

### Statistical analysis

The study cohort was divided into two groups according to the surgical technique: wedge resection versus lobectomy. The following variables were collected and considered for analysis: gender, age, clinical N/M stage, lung cancer pathology, pathological T/N stage, morbidity, and mortality. The clinical and pathological staging were reviewed and uniformly restaged according to the TNM VIII Edition (7). Quantitative variables were expressed as mean (standard deviation [SD]), whereas nominal variables were expressed binarily as the presence or absence of the event. Kruskal – Wallis Rank test was used for continuous variables and Fisher Exact test for categorical variables. Median OS and PFS were estimated by the reverse Kaplan – Meier method. Differences in survival rates were described by median OS, the hazard ratio (HR), and 95% confidence intervals (CI) and compared by the log-rank test. The stratified backward stepwise Cox regression model was employed for multivariable survival analyses. Backward elimination was performed with a p-value criterion of 0.20. The Akaike information criterion was used to estimate the models’ relative quality, selecting the ones with the best goodness of fit and avoiding collinearity bias. Significance was defined as a p-value <0.05. RStudio (R version 4.2.1, Funny-Looking Kid) was utilized for data analyses (8, 9). The standard, *EZR*, *irr*, and *rcmdr* packages were used for statistical analysis.

## Results

**Figure 1:**
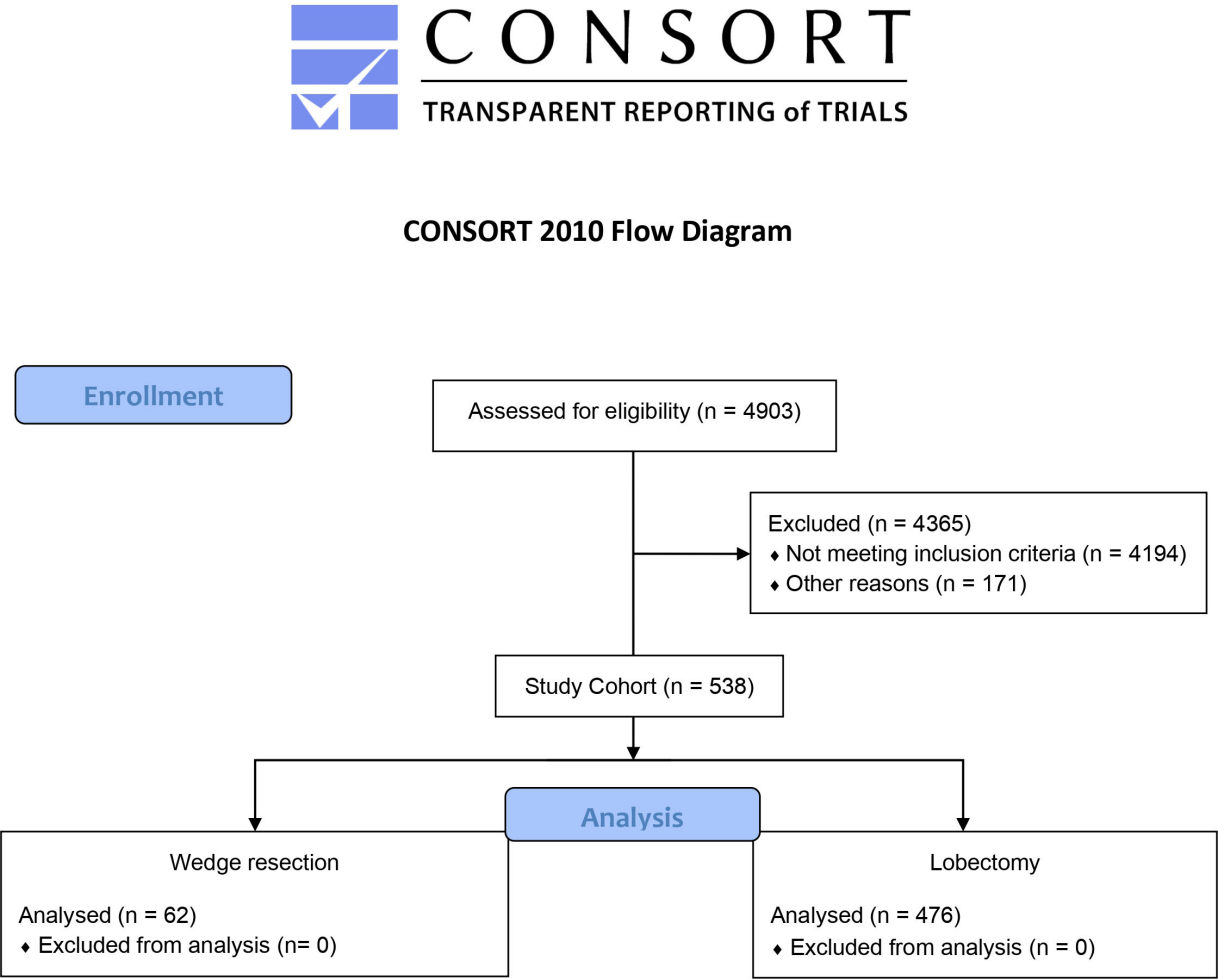
CONSORT flow diagram.

We assessed for eligibility 4903 clinical records of patients; we excluded 4365 patients (4194 patients did not meet the inclusion criteria, and 171 due to other reasons (e.g., data incompleteness). A total of 538 patients with cN0/cM0, stage IA NSCLC (≤20 mm) were identified (**Figure 1**). The treatment strategy was 476 (88.5%) lobectomies and 62 (11.5%) wedge resections. Wedge resections were executed due to reduced pulmonary function in 22 (35.5%) patients, comorbidities in 19 (30.6%) patients’ willingness in 11 (17.8%), and unspecified reasons in 10 (16.1%).

The baseline characteristics of the cohort are summarized in **Table 1**. There was no statistically significant difference between techniques in terms of age (p = 0.15), pulmonary respiratory function (p = 0.81 and p = 0.63, respectively, for Diffusion Lung Carbon Monoxide (CO)% and Forced Expiratory Volume in the first second), side of the disease (p = 0.86), lobe (p = 0.61). The most frequent histological subtype was adenocarcinoma in 450 (83.6%) patients, followed by squamous cell carcinoma in 75 (13.9%) patients. Nevertheless, there were no significant statistical differences regarding the distribution of the different histologies (p = 0.28) between approaches and the GGO rate (p = 0.61) between approaches. The upstage to pT2 was due to pleural invasion of the neoplasm in all cases, and to pT3 was due to multiple nodules in all cases. On the contrary, there were statistically significant differences between the surgical approaches in terms of postoperative staging: pT (p = 0.043), pN (p = 0.014), and pathological stage (p = 0.038). Mortality was absent for wedge resections and neglectable for lobectomies (1 [0.21%] patient). Postoperative complications were statistically significantly (p = 0.012) higher in lobectomies (overall 28.8%). The most frequent complication was atrial fibrillation (34%). The median postoperative length of hospital stay was significantly lower (p = 0.011) for the wedge resections (3.6 days, range 2.0 – 8.0 days) compared to lobectomies (6.0 days, range: 1 – 63 days).

**Table 1:** Characteristics of patients undergoing lobectomy and wedge resection for cT0/cM0 stage I lung cancer (<20 mm).

	Wedge No. = 62	Lobectomy No. = 476	*p-value*
**Age, mean (SD)**	69.9 (7.4)	64.0 (8.1)	*0.15*
**M/F ratio**	1.95	1.36	*0.45*
**FEV1%, mean (SD)**	81.6 (25.6)	94.1 (19.7)	*0.63*
**DLCO%, mean (SD)**	68.4 (20.7)	80.9 (20.4)	*0.81*
**cN0 [according to TNM VIII Ed (7)]**	62 (100)	476 (100)	*NA*
**cM0 [according to TNM VIII Ed (7)]**	62 (100)	476 (100)	*NA*
**Access** • **Thoracotomic** • **Robotic Assisted** • **Video-Assisted**	0 0 62 (100)	267 (56.1) 144 (30.3) 65 (13.7)	*0.0015*
**Side** • **Left** • **Right**	26 (41.9) 36 (58.1)	191 (40.1) 285 (59.9)	*0.86*
**Lobe** • **Right upper** • **Right middle** • **Right lower** • **Left upper** • **Left lower**	24 (38.7) 3 (4.8) 9 (14.5) 19 (30.6) 7 (11.4)	192 (40.4) 27 (5.7) 67 (14.1) 119 (25.0) 71 (14.8)	*0.61*
**GGOs** • **Pure GGO** • **Partially solid**	10 (16.1) 8 (12.9) 2 (3.2)	57 (12.0) 36 (7.6) 21 (4.4)	*0.61*
**Lung cancer pathology** • **Adenocarcinoma** • **Squamous cell carcinoma** • **Adenosquamous** **Large cell carcinoma**•	48 (77.4) 13 (21.0) 0 1 (1.6)	402 (84.5) 62 (13.1) 7 (1.5) 5 (1.1)0	*0.28*
**Size (mm), mean (SD)**	11.0 (4.2)	13.2 (4.3)	*0.12*
**pT [according to TNM VIII Ed (7)]** • **1 (is)** • **1a** • **1b** • **2a** • **2b** • **3**	1 (1.6) 41 (66.1) 13 (21.0) 5 (8.1) 1 (1.6) 1 (1.6)	1 (0.2) 138 (29.0) 298 (62.7) 10 (2.1) 17 (3.6) 12 (2.5)	*0.043*
**pN [according to TNM VIII Ed (7)]** • **x** • **0** • **1** • **2**	53 (85.5) 9 (14.5) 0 0	0 428 (89.9) 25 (5.3) 23 (4.8)	*0.014*
**p-Stage [according to TNM VIII Ed (7)]** • **IA1** • **IA2** • **IB** • **IIB** • **IIIA**	43 (69.3) 12 (19.4) 6 (9.7) 1 (1.6) 0	138 (29.0) 260 (54.6) 20 (4.2) 35 (7.3) 23 (4.8)	*0.038*
**Perioperative complications**	0	6 (1.2)	*NA*
**Postoperative complications**	2 (3.2)	137 (28.8)	*0.012*
**Mortality**	0	1 (0.21)	*NA*
**Length of hospital stay (day), median (range)**	3.6 (2.0 – 8.0)	6.0 (1 – 63)	*0.011*

DLCO%, Diffusion Lung Carbon Monoxide (CO); FEV1%, Forced Expiratory Volume in the first second; GGO, ground glass opacity; NA, not applicable; SD, standard deviation.

**Figure 2:**
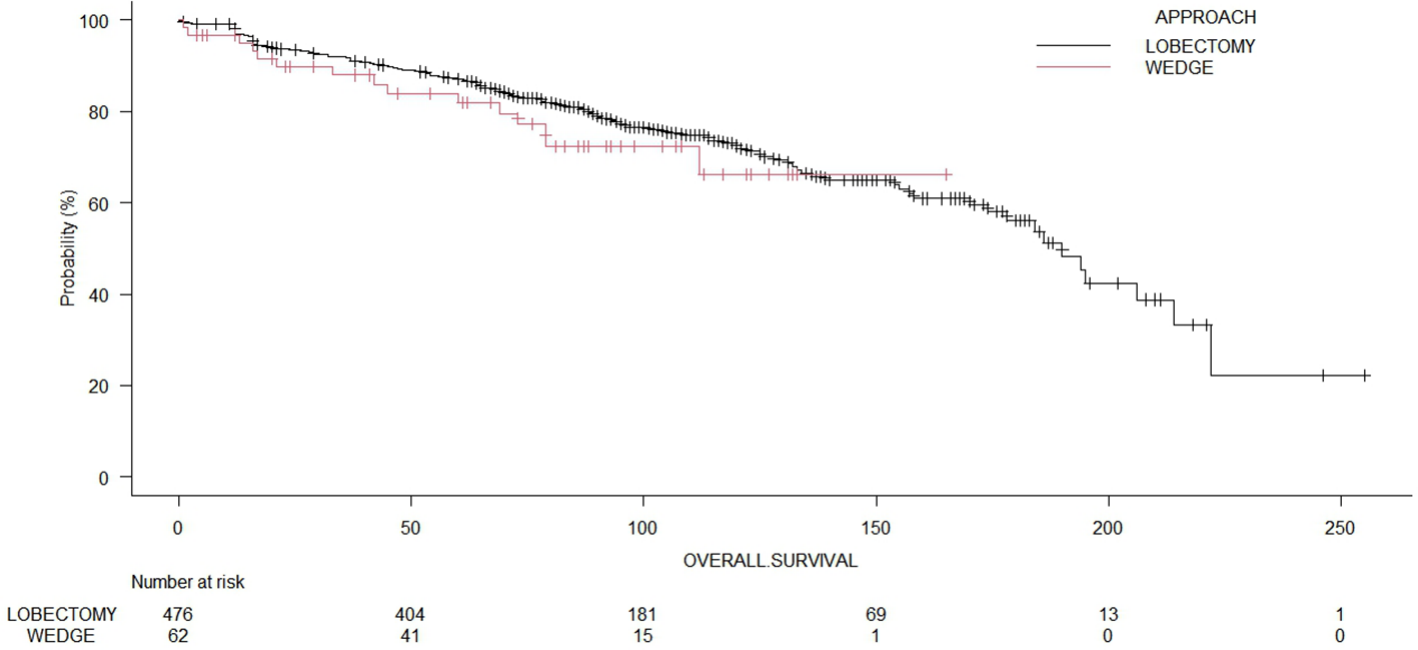
Comparison of overall survival of patients undergoing wedge resection or lobectomies (p = 0.39).

**Figure 3:**
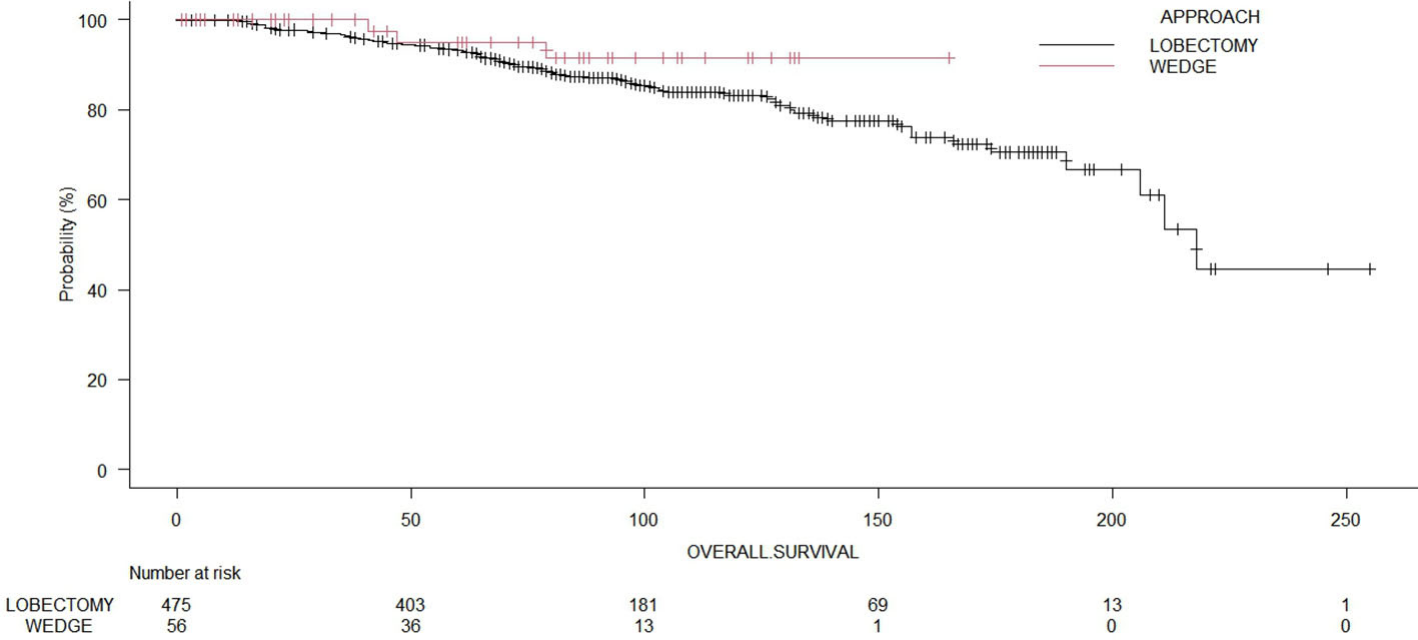
Comparison of recurrence-free survival of patients with wedge resection or lobectomies (p = 0.23).

The median OS time for the whole cohort was 190.0 months (range: 178 – 214 months). The 5-year OS of patients who underwent wedge resection and lobectomy were 82.2% and 87.0%, respectively. The cancer-specific survival for the whole cohort was 81.1%. The cancer-specific survival rates of patients who underwent wedge resection and lobectomy were 82.3% and 81.7%, respectively. The 5-year recurrence-free survival of patients who underwent wedge resection and lobectomy were 17.8% and 28.9%, respectively. The log-rank test showed no significant differences (p = 0.35) between wedge resections and lobectomies regarding OS (**Figure 2**). Even in terms of RFS (**Figure 3**), the log-rank test did not show significant differences (p = 0.25) between the surgical approaches. In a subanalysis of pathological stage IA comparing wedge resections and lobectomies, the log-rank test did not show significant differences (p = 0.16) between the surgical approaches (**Supplemental File 2**). **Table 2** presents the Cox proportional hazard regression results of RFS. Multivariate Cox regression analysis demonstrated that variables, including age, sex, DLCO%, side, site, and size, were not independent prognostic factors of RFS, while additionally, FEV1% was an independent prognostic factor of RFS (hazard ratio [HR] = 0.97; 95% confidence interval [CI]: 0.94 – 0.99, p = 0.019).

**Table 2:** Results of Cox regression analysis for progression-free survival.

Variable	HR	95%CI	p-value
**Age**	0.98	0.91 – 1.06	0.68
**Sex**	0.31	0.061 – 1.54	0.15
**FEV1%**	0.97	0.94 – 0.99	0.019
**DLCO%**	0.99	0.97 – 1.02	0.53
**Size**	0.99	0.84 – 1.18	0.92
**Side**	0.71	0.20 – 2.49	0.59
**Lobe**	0.62	0.30 – 1.30	0.20

CI, confidence interval; DLCO%, Diffusion Lung Carbon Monoxide (CO); FEV1%, Forced Expiratory Volume in the first second; HR, hazard ratio.

## Discussion

Although the incidence of small-sized lung adenocarcinoma should rise in this decade due to the widespread use of CT screening, the appropriate surgical treatment for these early-stage tumors is still unclear. Recent literature showed that limited resection could be a definitive treatment, and the argument is on which type of limited resection is still appropriate. Previous research evaluated the oncologic efficacy of lobectomy and sublobar resection without taking pathological subtyping into account and, more significantly, without excluding non-invasive patients (10). In clinical practice, wedge resection is always performed on patients with several comorbidities or poor lung function who may be unable to undergo a more thorough procedure. For patients with stage I NSCLC who can tolerate lobectomy, it was well established that lobectomy is superior to wedge resection (11). In a selected cohort of octogenarians with early-stage NSCLC tumors <2 cm, OS following wedge resection and lobectomy were comparable. Wedge resection was related to reduced toxicity, shorter operations, less operating blood loss, and fewer postoperative complications. In addition, the prevalence of other causes of death was lower following wedge resections than after anatomic resections.

Even if NSCLC <2 cm may have been less aggressive oncologically, the prognosis may be at least comparable between surgical methods since the OS was marginally better with wedge resection than after anatomic resection. For most patients undergoing wedge resection, lymph nodes were not collected. Even if the lymph nodes in the wedge resection group had been taken for pathologic staging, the preoperative clinical staging of the study population would not have changed. However, the global oncologic gold standard for all NSCLC resections involves lymph node dissection (12).

A systematic review and meta-analysis indicate that parenchymal-sparing and lobar resections have the same effect on survival for pT1a NSCLC. The optimistic outcomes of parenchymal-sparing surgery are due to its association with less lung volume loss and potentially less morbidity. The risk of nodal upstaging in cT1a parenchymal-sparing resections is a possible hazard, and the therapy of patients is poorly understood (13).

This analysis found that lobectomy is equivalent to wedge resection for early-stage NSCLC <20 mm. The JCOG0802 study reported the prognosis of segmentectomy and lobectomy for lung tumors less than 2 cm, suggesting that the segmentectomy had a greater local recurrence rate than lobectomy. However, the average tumor diameter in the JCOG0802 research was 1.6 cm, whereas this investigation focused on smaller lung nodules (3). Disagreeing with the findings of JCOG0802, the recurrence rate of segmentectomy was substantially equivalent (10).

Lymphadenectomy is a crucial component of lung cancer surgery as it helps determine the disease’s staging and prognosis. It involves the removal and examination of lymph nodes to assess the spread of cancer and guide postoperative treatment decisions. However, there is still no consensus on the optimal extent and technique of lymphadenectomy, and different guidelines and recommendations have been proposed by various societies of Thoracic Surgery and Oncology. Minimally invasive approaches to lymphadenectomy can achieve outcomes comparable to open surgery in terms of safety, feasibility, and effectiveness, particularly for the treatment of early-stage NSCLC. This implies that minimally invasive techniques can be considered viable alternatives to open surgery for lymphadenectomy (14).

Several studies have investigated the changes in postoperative lung function between lobectomy and segmentectomy, with some concluding that there is little difference between the two surgical methods in the maintenance of lung function. Other research, however, has identified significant differences. The distinctions between the surgical procedures regarding postoperative lung function preservation remain debatable. Moreover, pulmonary segmentectomy is gaining popularity due to its ability to maintain more lung tissue and enhance short-term results. Some retrospective studies have demonstrated that segmental pulmonary resection is comparable to lobectomy in terms of prognosis and local recurrence for small-stage IA NSCLC. The extent of surgical excision of early NSCLC remains disputed (15).

### Limitations

This research has a few drawbacks. First, this retrospective study utilized data from a single institution. Consequently, there may be a bias even if the institution was Italy’s most prominent cancer center. Secondly, most recurrence-suspected lesions were difficult to detect *via* biopsy and were evaluated primarily on clinical and radiological symptoms. Thirdly, most patients undergoing lobectomy may have had systematic lymph node dissection, whereas those undergoing wedge may not have had lymph node dissection or may have undergone lymph node sampling only due to their early stage or poorer physical status. Although our study focused on patients diagnosed with stage IA NSCLC, the number of negative lymph nodes removed between the resections may influence survival (16). Additionally, the database has no information regarding the postoperative quality of life. If the quality of life is significantly worse after surgical resection, patients will not benefit from any procedure, even if surgical resection entirely manages their cancer. Compared to wedge resection, lobectomy involves a greater awareness of reducing the postoperative quality of life. Lastly, we considered the number of wedges relatively small even if this study targeted a specific population to address the crucial clinical question of which surgical procedure is optimal for a subset of early-stage NSCLC.

## Conclusions

Our study demonstrates that lobectomy and wedge resection are equivalent oncologic treatments for individuals with cN0/cM0 stage IA NSCLC <20 mm. Validating the current findings will require a prospective, randomized comparison between wedge resection and standard lobectomy to establish the prognostic significance of wedge resection. The analysis of these data could aid in the formulation of clinical treatment recommendations and the planning of future clinical trials keeping well in mind the future role of bronchoscopic microwave ablation as an option in the treatment of malignant lung nodules (17).

## Data availability statement

The data analyzed in this study is subject to the following licenses/restrictions: The original contributions presented in the study are included in the article/**Supplementary Material**; further inquiries can be directed to the corresponding authors. Requests to access these datasets should be directed to luca.bertolaccini@gmail.com.

## Ethics statement

Ethical review and approval was not required for the study on human participants in accordance with the local legislation and institutional requirements. Written informed consent for participation was not required for this study in accordance with the national legislation and the institutional requirements.

## Author contributions

LB and LS contributed to the conception and design of the study. DB, RB, and GS organized the database. LB performed the statistical analysis. LB wrote the first draft of the manuscript. AC, MC, CD, NG, AM, SM, and CU wrote sections of the manuscript. All authors contributed to the article and approved the submitted version.
