# Supplementary figures and images for: Real-world survival outcomes of wedge resection versus lobectomy for cT1a/b cN0 cM0 non-small cell lung cancer: a single center retrospective analysis
Source: Front Oncol. 2023 Aug 17;13:1226429. doi: 10.3389/fonc.2023.1226429 (PMC10470827; doi:10.3389/fonc.2023.1226429)

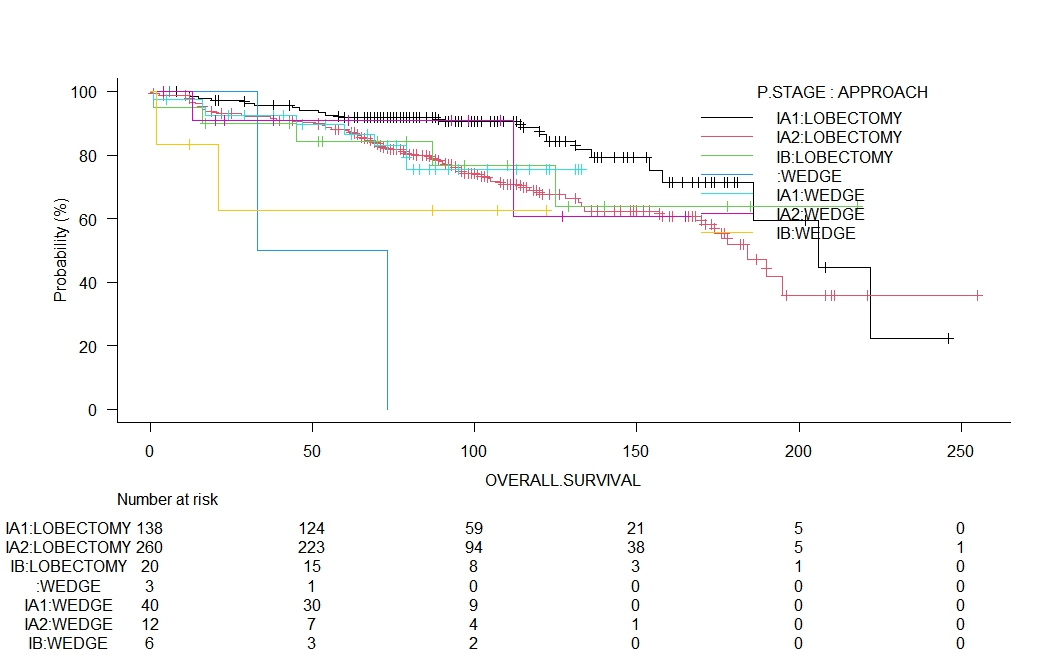

Supplement: Supplementary file 1 [file Image_1.jpeg]
